# Headache as a Predictor of Cryptococcal Meningitis in Ambulatory Patients With Symptomatic HIV-associated Cryptococcal Antigenemia

**DOI:** 10.1093/ofid/ofag027

**Published:** 2026-01-20

**Authors:** Sarah M Najjuka, Alexandra Poeschla, Abduljewad Wele, Elizabeth Nalintya, Paul Kirumira, Peruth Ayebale, Grace Nakitto, Fred Turya, Lydia Nankungu, Caleb P Skipper, Ann Fieberg, Biyue Dai, David R Boulware, David B Meya, Radha Rajasingham

**Affiliations:** Infectious Diseases Institute, College of Health Sciences, Makerere University, KampalaUganda; Division of Infectious Diseases & International Medicine, Department of Medicine, University of Minnesota, Minneapolis, Minnesota, USA; Division of Biostatistics and Health Data Science, University of Minnesota, Minneapolis, MinnesotaUSA; Infectious Diseases Institute, College of Health Sciences, Makerere University, KampalaUganda; Infectious Diseases Institute, College of Health Sciences, Makerere University, KampalaUganda; Infectious Diseases Institute, College of Health Sciences, Makerere University, KampalaUganda; Infectious Diseases Institute, College of Health Sciences, Makerere University, KampalaUganda; Infectious Diseases Institute, College of Health Sciences, Makerere University, KampalaUganda; Infectious Diseases Institute, College of Health Sciences, Makerere University, KampalaUganda; Division of Infectious Diseases & International Medicine, Department of Medicine, University of Minnesota, Minneapolis, Minnesota, USA; Division of Biostatistics and Health Data Science, University of Minnesota, Minneapolis, MinnesotaUSA; Division of Biostatistics and Health Data Science, University of Minnesota, Minneapolis, MinnesotaUSA; Division of Infectious Diseases & International Medicine, Department of Medicine, University of Minnesota, Minneapolis, Minnesota, USA; Infectious Diseases Institute, College of Health Sciences, Makerere University, KampalaUganda; Division of Infectious Diseases & International Medicine, Department of Medicine, University of Minnesota, Minneapolis, Minnesota, USA; Division of Infectious Diseases & International Medicine, Department of Medicine, University of Minnesota, Minneapolis, Minnesota, USA

**Keywords:** advanced HIV disease, cryptococcal antigenemia, cryptococcosis

## Abstract

**Background:**

People with HIV-associated cryptococcal antigenemia are at high risk for meningitis and death. In resource-limited settings, lumbar puncture to evaluate for meningitis is infrequently performed in asymptomatic individuals; therefore, symptomatic individuals are prioritized. We evaluated the predictive value of meningeal symptoms for cryptococcal meningitis in people with cryptococcal antigenemia.

**Methods:**

We conducted a secondary analysis from 3 randomized clinical trials conducted in Uganda between 2017 and 2024. We included adults with meningeal symptoms who had cerebrospinal fluid cryptococcal antigen (CrAg) testing performed. Logistic regressions and classification trees were used to determine the associations between meningeal symptoms and meningitis. Area under the receiver operating characteristic curve (AUROC) and misclassification rate were used to evaluate model performance.

**Results:**

Among 344 participants, median CrAg titer was 1:1280 (interquartile ratio, 1:100-1:2560). Overall, 285 (83%) presented with headache and 205 (60%) participants had meningitis. Presence of headache was associated with meningitis (adjusted odds ratio 11.7; 95% confidence interval [CI], 5.23-28.50). The AUROC of headache alone to predict meningitis was 0.65 (95% CI, 0.61-0.69), with 95% sensitivity and 35% specificity. The AUROC improved to 0.86 (95% CI, 0.82-0.90) when stiff neck, photophobia, confusion, sex, and CrAg titer ≥1:160 were added to the logistic regression model. In the final classification tree, headache combined with CrAg titer ≥1:160 demonstrated the highest probability (79%) of meningitis.

**Conclusions:**

Headache and high CrAg titer is the most reliable predictor for meningitis. In the absence of plasma CrAg titration, symptoms of headache, stiff neck, photophobia, and confusion predict meningitis for individuals with cryptococcal antigenemia.

## Teaser

The presence of headache and high CrAg titer is the most reliable predictor for meningitis. In the absence of plasma CrAg titration, a combination of symptoms including headache, stiff neck, photophobia, and confusion are most indicative of meningitis.

Cryptococcal meningitis is the most common cause of HIV-related meningitis and is estimated to cause 19% of AIDS-related deaths worldwide [1]. Subclinical infection, known as cryptococcal antigenemia, can be identified by detection of cryptococcal antigen (CrAg) in the bloodstream before onset of fulminant meningitis [2]. The World Health Organization recommends screening all people with HIV and CD4 count <100 cells/µL for CrAg in the blood, and subsequent fluconazole preemptive treatment for those who are CrAg positive to mitigate the risk of meningitis and death [3]. Given that ∼30% of blood CrAg-positive people will go on to develop fulminant meningitis, lumbar puncture (LP) is recommended by some national guidelines [4]. Practically, because of limited resources in busy clinics and patient fears of LPs, LPs are often only performed in symptomatic individuals [5, 6], assuming that symptoms are suggestive of meningitis.

High plasma CrAg titer is a risk factor for meningitis and death [7, 8]. However, symptoms such as headache that are commonly associated with meningitis have not been thoroughly evaluated as indicators of cryptococcal meningitis among persons with cryptococcal antigenemia. In the clinical setting, people with HIV who are CrAg positive are typically screened for symptoms including headache, fever, neck stiffness, and neurologic changes. People reporting symptoms may be triaged for LP, but it is unknown which symptoms in particular are predictive of meningitis in people with cryptococcal antigenemia.

The objective of this study was to evaluate which symptoms are predictive of meningitis among people with HIV-associated cryptococcal antigenemia. Understanding the symptomatology associated with meningitis among people with cryptococcal antigenemia would allow healthcare workers to provide better counseling for LPs.

## METHODS

### Study Design, Site, and Population

This is a secondary analysis of data from 3 randomized clinical trials; C-ASSERT (clinicaltrials.gov NCT03002012), ORCAS/ACACIA (NCT03945448), and the ENCORE (NCT05085171) trials conducted in Uganda between November 2017 and August 2024. In this analysis, we included adult (≥18 years) outpatient participants with advanced HIV who were plasma CrAg positive with an LP performed and cerebrospinal fluid (CSF) CrAg result. All participants had clinical assessment for symptoms of meningitis within 7 days of the LP. These included presence of headache, fever, photophobia, visual changes, seizures, confusion, mania, and Glasgow Coma Scale < 15. All CrAg testing (CSF and plasma) was performed using the CrAg lateral flow assay (LFA) (Immy, Norman, OK).

### Study Variables

The primary outcome of interest was a CSF CrAg-positive result indicating cryptococcal meningitis. Baseline characteristics of interest include symptoms presentation, specifically presence of headache, neck stiffness, fever, confusion, visual disturbances, and focal neurologic deficits. Additionally, baseline CD4 cell count, plasma CrAg titer, antiretroviral therapy (ART) status (naive or experienced) and duration on ART were summarized. Baseline laboratory values were collected including serum creatinine, sodium, potassium, hemoglobin, and platelet count.

### Statistical Analysis

Baseline characteristics and laboratory characteristics were summarized for the overall cohort, as well as by CSF CrAg test results. Continuous variables were summarized using medians and interquartile ranges (IQR). Categorical variables, including presence of signs and symptoms, were summarized using frequencies and percentages. Statistical comparisons between the CSF CrAg-positive and CSF CrAg-negative groups were made using the Kruskal-Wallis test for medians and chi-squared test or Fisher exact test for proportions. CrAg titer was categorized using a cutoff of 1:160, based on prior evidence showing that patients with a titer of 1:≥160 have a 3-fold increased risk of death than those with low titers (<1:160) [9, 10]. The overall distribution of each meningeal sign and symptom was summarized.

For symptoms that were associated with CSF CrAg status in the univariate categorical analysis with a *P* value less than a prespecified cutoff 0.10, unadjusted and adjusted odds ratios were obtained via univariate and multivariable logistic regression models to evaluate the magnitude of their association with positive CSF CrAg results. Baseline characteristics and clinical variables were adjusted in the multivariable models to account for confounding. Model selection for the full multivariable model was conducted via a backward stepwise algorithm based on Akaike Information Criterion. Areas under the receiver operating characteristic curves (AUROC) were reported to evaluate the discriminating ability of the signs and symptoms for positive CSF CrAg results. DeLong's method was used to compare ROC curves from different models and to construct confidence intervals for the AUC statistics.

Continuous variables with missing values were imputed with the medians of the complete data. As sensitivity analysis, we used the Multiple Imputation by Chained Equations. Using the mice package in R [11], we generated 15 complete data sets. Then a logistic regression for the predicting positive LP was performed on each of the 15 complete datasets. The area under the curve (AUC) for each model was calculated. The resulting odds ratio estimates, AUC values, and their associated variances were then pooled using Rubin's rules [12]. The final pooled AUC estimate was then used to generate the presented performance ROC plot.

To assess the accuracy and performance of the logistic regression models, the dataset was randomly partitioned into training and testing sets at a 4:1 ratio. A model was fitted to the training set to obtain model estimates, and the optimal probability cutoff was determined using the threshold that maximized the Youden Index. The resulting model and probability cutoff was then applied to the test set. Using data from the test set, calibration plots with calibration intercepts and slopes were generated using R package CalibrationCurves [13, 14]. An additional ROC curve was also constructed only using the test dataset. Positive predictive value and negative predictive value of the optimal probability cutoff was assessed over outcome prevalences using Bayes’ Rule [15]. A classification tree was constructed using the Classification And Regression Tree algorithm with the rpart R package in R version 4.3.2 (R Foundation for Statistical Computing, Vienna, Austria) [16]. The dataset was partitioned into a training set and a test set, with an 80% to 20% ratio. The classification tree was first fitted on the training set, where the default 10-fold cross-validation error was used for model pruning and to protect against overfitting. After the final pruned model was selected, the independent test set was then used to calculate the misclassification rate. Additional model calibrations and evaluations were also conducted on the test set.

### Ethical Considerations

The clinical trials from which data for this study was extracted conformed to the U.S. and international standards of Good Clinical Practice, the Declaration of Helsinki, and international ethical guidelines for biomedical research involving human subjects. They were also conducted in compliance with the relevant government regulations for Uganda, the United States, and international and institutional research policies and procedures. All study protocols were approved by relevant research and ethics committees, the Uganda National Council for Science and Technology, Uganda National Drug Authority, and the University of Minnesota. All participants provided written informed consent before enrollment into the studies and the funder had no role in the study conduct or data analysis. Study data were accessible to all the authors of this study.

## RESULTS

Of 365 serum CrAg-positive participants who had CSF CrAg testing performed, 344 had complete data for the presence or absence of symptoms of meningitis within 7 days of lumbar puncture and were included in this analysis. The median age of participants was 36 years (IQR 30, 41), and 45% were female (Table 1). Overall, 65% were ART-naive, and the median CD4 cell count was 33 cells/µL (IQR 16, 68). The median CrAg titer was 1:1280 (IQR 1:100, 1:2560). Headache was the most common presenting symptom (83%), followed by fever (35%), stiff neck (33%), photophobia (23%), and confusion (14%) (Supplementary Figure 1). Baseline characteristics of participants who were excluded are summarized in Supplementary Table 1.

**Table 1.** Baseline Characteristics of Participants by CSF CrAg Outcome

Variable	N With Data	Overall Cohort	Positive CSF CrAg	Negative CSF CrAg	*P* Value
No. per group	344	344	205	139	
Demographics					
Age, y	344	36 [30, 41]	36 [30, 41]	36 [30, 42]	.77
Male	344	188 (55%)	109 (58%)	79 (48%)	<.01
Antiretroviral therapy (ART)					
Currently on ART	96	96 (28%)	51 (25%)	45 (32%)	.31
Never on ART	223	223 (65%)	139 (68%)	84 (60%)	
Previously on ART	25	25 (7%)	15 (7%)	10 (7%)	
Duration on ART, m	94	1.6 [0.4, 6.7]	1.7 [0.5, 4.5]	1.6 [0.4, 11.5]	.87
Signs and symptoms ^a^					
Glasgow Coma Scale <15	283	27 (10%)	18 (10)	9 (9%)	.67
Headache	344	285 (83%)	195 (95%)	90 (65%)	<.001
Stiff neck	344	115 (33%)	94 (46%)	21 (15%)	<.001
Fever	344	122 (36%)	74 (36%)	48 (34%)	.77
Mania	344	18 (5%)	11 (5%)	7 (5%)	.89
Confusion	344	48 (14%)	35 (17%)	13 (10%)	.04
Seizures	344	17 (5%)	14 (7%)	3 (2%)	.05
Photophobia	344	79 (23%)	64 (31%)	15 (11%)	<.001
Focal neurologic deficits	344	11 (3%)	5 (2%)	6 (4%)	.33
Visual changes	312	14 (5%)	7 (4%)	7 (6%)	.41
Other CNS symptoms	344	65 (19%)	36 (18%)	29 (21%)	.44
Baseline laboratory results					
CD4+ cell count/µL	242	33 [16, 68]	28 [15, 56]	38 [19, 79]	.07
Plasma CrAg titer, 1:x	278	1280 [100, 2560]	2560 [640, 2560]	80 [20, 640]	<.001
Plasma CrAg titer ≥1:160	344	208 (60%)	152 (74%)	56 (40%)	<.001
Sodium, mmol/L	115	134 [130, 137]	132 [127, 134]	134 [131, 137]	<.01
Hemoglobin, g/dL	118	11.3 [9.9, 12.7]	11.7 [10.4, 13.0]	11.3 [9.9, 12.4]	.47
Potassium, mmol/L	115	4.3 [4.0, 4.6]	4.0 [3.6, 4.3]	4.3 [4, 4.6]	<.01
Platelets ×10^3^/µL	113	261 [187, 343]	252 [171, 335]	269 [187, 343]	.62
Creatinine, mg/dL	114	0.7 [0.6, 0.9]	0.6 [0.6, 0.7]	0.7 [0.6, 0.9]	.12

Data are presented as n (%) or median [IQR].

Abbreviations: ART, antiretroviral therapy; CrAg, cryptococcal antigen; IQR, interquartile range.

^a^Symptoms were assessed within ± 7 days from the date of the lumbar puncture.

Overall, 60% (205/344) of participants were CSF CrAg-positive. Among CSF CrAg-positive participants, the median plasma CrAg titer was 1:2560 (IQR 1:640, 1:2560), whereas among CSF CrAg-negative participants, the median plasma CrAg titer was 1:80 (IQR 1:20, 1:640) (Table 1). Participants who were CSF CrAg-positive were more likely to report a baseline headache (95% vs 65%, *P* < .001), stiff neck (46% vs 15%, *P* < .001), photophobia (31% vs 11%, *P* < .001), or confusion (17% vs 10%, *P* = .04). All 4 symptoms were positively associated with CSF CrAg-positive status in both the univariate model and multivariable logistic regression models, indicating that presence of any of these symptoms would increase the odds of having a CSF CrAg-positive result (Table 2).

**Table 2.** Univariate and Multivariable Logistic Regression Models

Univariate Models			
Symptoms	OR (95% CI)	*P* Value	AUC (95% CI)
Headache	10.6 (5.35–23.1)	<.001	0.65 (0.61–0.69)
Stiff neck	4.76 (2.82–8.33)	<.001	0.65 (0.61–0.70)
Photophobia	3.75 (2.08–7.14)	<.001	0.60 (0.56–0.64)
Confusion	2.00 (1.04–4.06)	.045	0.54 (0.50–0.57)
Clinical characteristics			
Male sex	1.98 (1.28–3.08)	.002	0.58 (0.53–0.64)
Crag titer ≥ 1:160	17.0 (8.45–38.3)	<.001	0.70 (0.65–0.74)

Multivariable model with symptoms alone			
Symptoms	Adjusted OR (95% CI)	*P* Value	AUC (95% CI)
Headache	12.6 (5.83–30.2)	<.001	0.78 (0.74–0.82)
Stiff neck	3.78 (2.07–7.20)	<.001	
Photophobia	2.34 (1.20–4.79)	.015	
Confusion	2.67 (1.11–7.02)	.035	

Multivariable full model			
Symptoms	Adjusted OR (95% CI)	*P* Value	AUC (95% CI)
Headache	11.7 (5.23–28.50)	<.001	0.86 (0.82–0.90)
Stiff neck	3.12 (1.60–6.34)	.001	
Photophobia	2.04 (0.98–4.49)	.065	
Confusion	2.41 (0.89–7.31)	.100	
Clinical characteristics		…	
Male sex	1.75 (1.00–3.09)	.052	
CrAg titer ≥1:160	12.2 (5.78–28.50)	<.001	

Abbreviations: AUC, area under the curve; CI, confidence interval; CrAg, cryptococcal antigen; OR, odds ratio.

**Figure 1.**
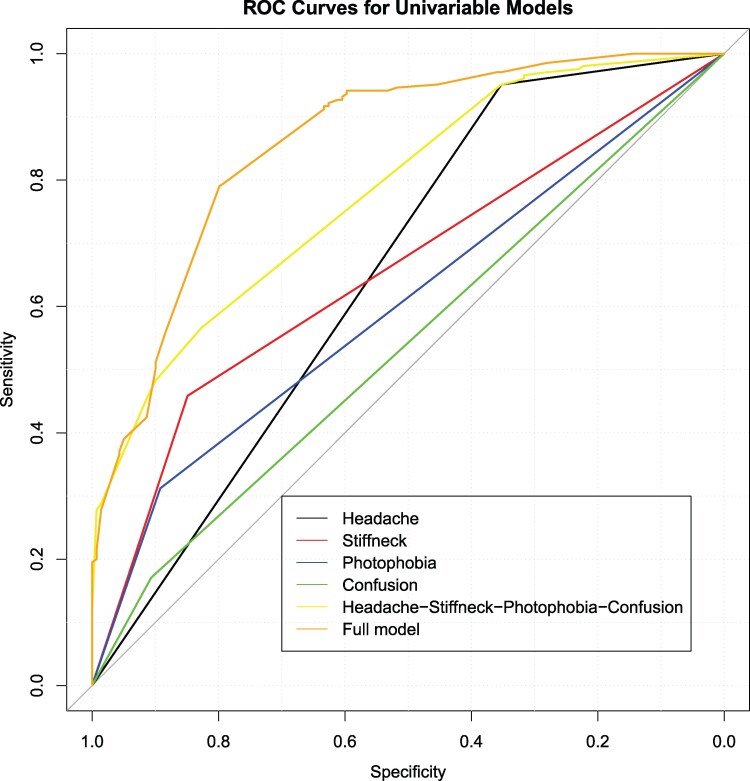
ROC curves for univariate analysis along with full model. Area under the receiver operating characteristic curves (AUROC) for univariate along with multivariate analysis. Headache has the highest sensitivity of about 0.95 and lowest specificity of about 0.36. Other symptoms when considered in isolation are less sensitive but highly specific for meningitis. A combination of all 4 symptoms increases the specificity compared to headache alone. The full model further increases the specificity at the same sensitivity of headache alone.

Univariate AUC statistics of headache alone was 0.65 (95% confidence interval [CI], .61–.69), indicating that the predictive accuracy of headache alone for a positive CSF CrAg result was poor; headache was 95% sensitive and only 35% specific for predicting meningitis. However, the overall AUC increased to 0.78 (95% CI, .74–.82) when all four symptoms (headache, neck stiffness, photophobia, confusion) were combined in a multivariable model (DeLong's test *P* value < .001) (Figure 1). The AUC was further improved to 0.86 (95% CI, .82–.90) when additional clinical characteristics of male sex and CrAg titer ≥1:160 were included in the model.

**Figure 2.**
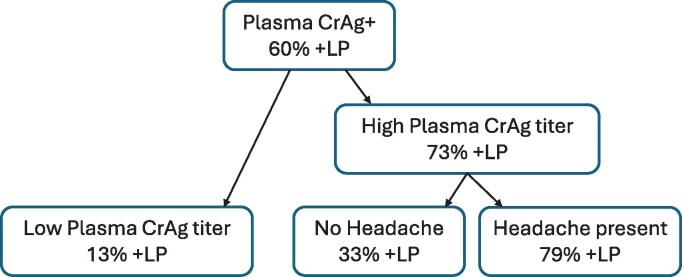
Classification tree for full model. The final pruned model, with a misclassification rate of 0.21, consists of 2 splits, with the first split by plasma CrAg titer status and second split by headache among participants with high plasma CrAg titer. Participants who had both high plasma CrAg titer and headache, which consists of 67% of the cohort, had the highest probability (79%) for having a CSF CrAg-positive result in the final pruned model, whereas the probability for having a CSF CrAg-positive result is only 13% and 33% in the other 2 groups. +LP in the figure denotes CSF CrAg-positive.

The variables used in the full multivariable logistic regression model were further evaluated using a classification tree. The final pruned model, with a misclassification rate of 0.21, consists of 2 splits, with the first split by plasma CrAg titer status and the second split of headache among those with high plasma CrAg titer (Figure 2). Participants who had high plasma CrAg titer ≥1:160 and headache had a 79% probability of a CSF CrAg-positive result in the final pruned model compared to the 18% probability for participants who had headache and a low CrAg titer ≤1:160.

Participants with a positive CSF CrAg had lower serum sodium levels (hyponatremia) compared to those who had a negative CSF CrAg. There were no significant differences in other laboratory findings among the two populations. Please see Supplementary Figures 2–9 for results from multiple imputation analysis, calibration plots, and model valuations.

## DISCUSSION

We assessed the predictive value of meningeal symptoms for cryptococcal meningitis in an ambulatory population with symptomatic cryptococcal antigenemia. In our cohort, where 60% of participants had meningitis (CSF CrAg-positive), headache was the most common presenting symptom. Having a combination of headache, stiff neck, photophobia, confusion, male sex, and CrAg titer ≥1:160 demonstrated the highest AUC. A combination of headache and CrAg titer ≥1:160 demonstrated a 79% probability of meningitis using a classification tree.

Similar to our study findings, a study conducted at 2 national referral hospitals in Uganda indicated that 56% of people with HIV-associated cryptococcal antigenemia had meningitis [17], although this study was conducted in an inpatient population. Headache is a common complaint prompting people to seek medical care [18]; it is also one of the most frequent symptoms of cryptococcal meningitis with prevalence above 75% [18–20]. This is consistent with the current study that identified headache in 83% of participants. Despite the high sensitivity of headache for predicting cryptococcal meningitis, headache alone is nonspecific for predicting meningitis [21].

The addition of stiff neck, photophobia, and confusion increased the specificity of headache and improved the overall AUC. When compared to the univariate headache model, the improvement in the AUC of the full model, which includes symptoms, male sex, and CrAg titer ≥1:160, was greatly attributed to the improved specificity from about 0.36 to 0.60 at the threshold that leads to the same 0.95 sensitivity. The combination of headache and high plasma CrAg titer had the highest probability of finding a positive CSF CrAg in our study, according to the classification tree model. This is consistent with findings by Wake and colleagues that suggested that a combination of headache and high blood CrAg titer highly predict a positive CSF CrAg [8]. In addition, high plasma CrAg titer alone has been associated with a positive CSF CrAg and death among those with a positive plasma CrAg [22, 23].

CrAg titers can be determined by a serial dilution technique using CrAg LFA, a process that requires technical expertise and with a relative long turnaround time for clinical decision making [24]. A rapid, point-of-care CrAg semiquantitative LFA presents a cost-effective alternative with the ability to determine CrAg titers in real time [25]. However, both methods of plasma CrAg titer determination are still widely inaccessible and may only be available in clinical trial settings, which presents a challenge in ambulatory HIV care settings where most patients with cryptococcal antigenemia initially present. Where CrAg titers are unavailable, clinicians should consider presence of the 4 symptoms (ie, headache, neck stiffness, photophobia, and confusion) to inform their decision to perform a lumbar puncture for those with a positive plasma CrAg, rather than considering headache in isolation.

In our study, participants with a positive CSF CrAg had lower serum sodium levels (hyponatremia) compared to those who had a negative CSF CrAg. Hyponatremia has been reported as a predictor of *Cryptococcal meningitis* and death among asymptomatic persons with *Cryptococcal antigenemia* and should inform clinicians of the increased risk of morbidity and mortality in this ambulatory population [26].

Limitations of our analysis are related to the retrospective nature of this study. We included participants enrolled in clinical trials, which excludes those who were too sick for study participation. Additionally, we included only participants who had lumbar punctures performed; those who declined or who died before having an LP were excluded from the study. This resulted in an unusually high prevalence of meningitis (60%), which limits generalizability of our findings. Furthermore, asymptomatic CrAg-positive participants were also excluded. Additionally, headache is a subjective experience, and reporting is dependent on multiple factors, including severity, presence of other symptoms, manner of asking, and documentation. It is therefore difficult to ascertain whether the reported absence of headache in individuals with symptoms like neck stiffness, photophobia, or confusion can be relied on.

In summary, headache alone is a nonspecific predictor of meningitis in persons with cryptococcal antigenemia in the outpatient setting. The presence of both headache and a high CrAg titer serves as the most reliable predictor for meningitis. In the absence of plasma CrAg titration, clinicians should consider a combination of symptoms such as headache, stiff neck, photophobia, and confusion, along with male sex, when deciding which patients to prioritize to perform a lumbar puncture to exclude cryptococcal meningitis among individuals with cryptococcal antigenemia.

## Supplementary Material

ofag027_Supplementary_Data

## Supplementary Data

Supplementary materials are available at *Open Forum Infectious Diseases* online. Consisting of data provided by the authors to benefit the reader, the posted materials are not copyedited and are the sole responsibility of the authors, so questions or comments should be addressed to the corresponding author.
