# Supplementary material for: Headache as a Predictor of Cryptococcal Meningitis in Ambulatory Patients With Symptomatic HIV-associated Cryptococcal Antigenemia
Source: Open Forum Infect Dis. 2026 Jan 20;13(2):ofag027. doi: 10.1093/ofid/ofag027 (PMC12888811; doi:10.1093/ofid/ofag027)
Supplement: ofag027_Supplementary_Data [file ofag027_supplementary_data.docx]

**Supplemental Table 1**

Baseline characteristics of included and excluded participants

| **Characteristic** | **N** | **Overall**  N = 365 | **Excluded**  N = 21 | **Included**  N = 344 | **p-value** |
| --- | --- | --- | --- | --- | --- |
| Age in Years | 365 | 36 (30, 41) | 39 (32, 45) | 36 (30, 41) | 0.213 |
| Female | 365 | 164 (45%) | 8 (38%) | 156 (45%) | 0.517 |
| ART Status | 364 |  |  |  | 0.095 |
| Currently on ART |  | 102 (28%) | 6 (30%) | 96 (28%) |  |
| Never On ART |  | 233 (64%) | 10 (50%) | 223 (65%) |  |
| Previously On ART |  | 29 (8.0%) | 4 (20%) | 25 (7.3%) |  |
| Months on ART | 100 | 2 (0, 11) | 23 (0, 26) | 2 (0, 7) | 0.523 |
| GCS | 363 |  |  |  | <0.001 |
| < 15 |  | 99 (27%) | 17 (81%) | 82 (24%) |  |
| = 15 |  | 264 (73%) | 4 (19%) | 260 (76%) |  |
| Headache | 365 | 299 (82%) | 14 (67%) | 285 (83%) | 0.077 |
| Stiff neck | 365 | 115 (32%) | 0 (0%) | 115 (33%) | 0.001 |
| Fever | 365 | 128 (35%) | 6 (29%) | 122 (35%) | 0.520 |
| Mania | 365 | 18 (4.9%) | 0 (0%) | 18 (5.2%) | 0.613 |
| Confusion | 365 | 49 (13%) | 1 (4.8%) | 48 (14%) | 0.333 |
| Seizures | 365 | 17 (4.7%) | 0 (0%) | 17 (4.9%) | 0.612 |
| Photophobia | 365 | 79 (22%) | 0 (0%) | 79 (23%) | 0.011 |
| Focal neuro Deficit | 365 | 14 (3.8%) | 2 (9.5%) | 12 (3.5%) | 0.189 |
| Visual- change | 365 | 17 (4.7%) | 1 (4.8%) | 16 (4.7%) | >0.999 |
| Other CNS symptoms | 365 | 70 (19%) | 5 (24%) | 65 (19%) | 0.571 |
| Blood CrAg Titer | 301 |  |  |  | 0.588 |
| CrAg Titer<160 |  | 77 (26%) | 6 (32%) | 71 (25%) |  |
| CrAg Titer>=160 |  | 224 (74%) | 13 (68%) | 211 (75%) |  |

1 Median (Q1, Q3); n (%)

2 Wilcoxon rank sum test; Pearson’s Chi-squared test; Fisher’s exact test

**Supplemental Figure 1:** Percentage distribution of signs and symptoms of meningitis among participants (N=344). Headache was the most common symptom followed by fever, stiff neck, and photophobia.

**Supplemental Table 2**

Pooled Model estimates using Multiple Imputation

| **Characteristic** | **OR (95% CI)** | **p-value** |
| --- | --- | --- |
| Gender (Male) | 1.86 (1.04, 3.31) | 0.035 |
| Headache | 11.36 (4.77, 27.06) | <0.001 |
| CrAg Titer >= 160 | 11.94 (5.30, 26.90) | <0.001 |
| Stiff Neck | 3.13 (1.56, 6.30) | 0.001 |
| Photophobia | 2.01 (0.92, 4.42) | 0.080 |
| Confusion | 2.62 (0.90, 7.67) | 0.078 |

**Supplemental Figure 2**

Pooled ROC with multiple Imputation

**
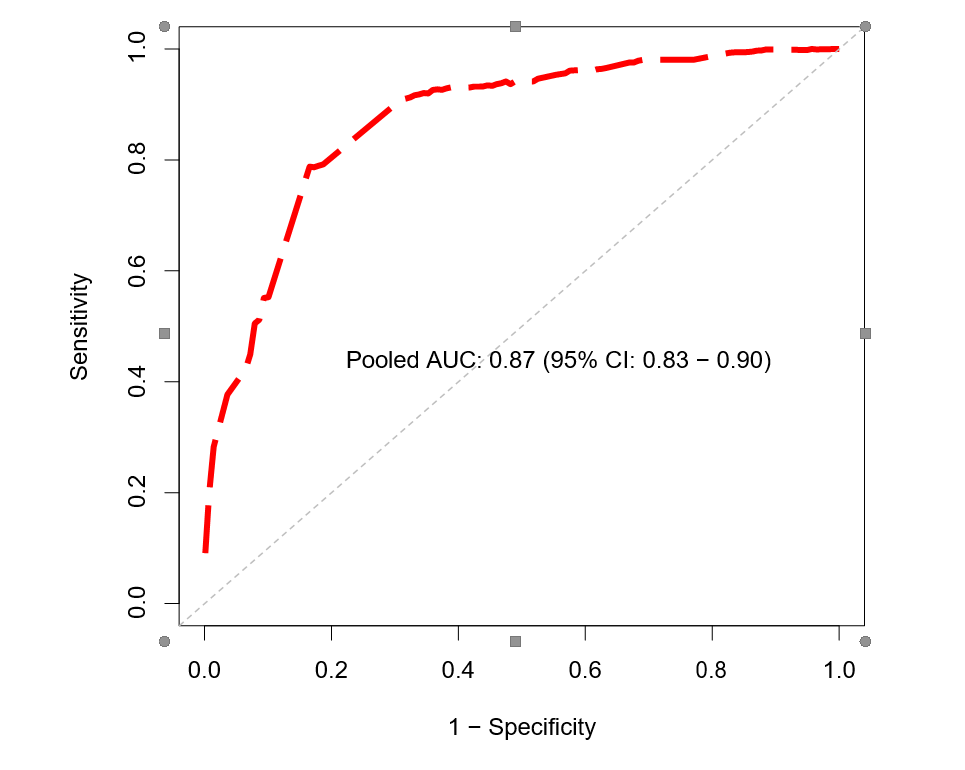
**

**Supplemental Table 3**

Model estimates from the training set

| **Characteristic** | **OR (95% CI)** | **p-value** |
| --- | --- | --- |
| Gender(Male) | 2.24(1.21, 4.19 4.19) | 0.011 |
| Headache | 8.77(3.65, 23.3 23.3) | <0.001 |
| CrAg Titer >= 160 | 9.83(4.40, 24.4 24.4) | <0.001 |
| Stiffneck | 3.44(1.65, 7.59 7.59) | 0.001 |
| Photophobia | 1.67(0.77, 3.73 3.73) | 0.2 |
| Confusion | 2.58(0.81, 9.43 9.43) | 0.13 |

**Supplemental Figure 3**

0.8

1.0

A

UC:0

.89(0

.816–

0.974

)

1.0 0.8 0.6 0.4 0.2 0.0

0.0

0.2

Specificity

**Supplemental Figure 4:** PPV and NPV vs Prevalence for the full logistic regression model at the optimal probability cutoff that maximizes Youden Index in training set.

1.00

Sens =

0.951, S

ec =0.7

0, Prob T

hrehold

s =0.60

p

5

0.75

0.50

Values

0.25

0.00

0.0 0.2 0.4 0.6 0.8

## Prevalence

Metric NPV PPV

**Supplemental Figure 5:** Calibration Plot for the logistic regression

1.0

Calibration

...intercept: 0.13 (−0.48 to 0.75)

...slope: 1.28 (0.71 to 1.85)

Discrimination

...c−statistic: 0.90 (0.82 to 0.97)

Ideal

Logistic calibration Flexible calibration (Loess)

1

0

0.8

0.6

Observed proportion

0.4

0.2

0.0

0.0 0.2 0.4 0.6 0.8 1.0

Predicted probability

**Supplemental Figure** **6:** Checking PPV and NPV for Tree Model

Sensitivity

0.4

0.6

0.8

1.0

AUC:

0.841

(0.75

–0.9

1)

2

3

1.0 0.8 0.6 0.4 0.2 0.0

0.0

0.2

Specificity

**Supplemental Figure 7**. PPV and NPV vs Prevalence for the tree model at the optimal probability cutoff that maximizes Youden Index in training set

1.00

Sen

s =0.976,

Spec =0

.714

0.75

0.50

Values

0.25

0.00

0.0 0.2 0.4 0.6 0.8

## Prevalence

Metric NPV PPV

**Supplemental Figure 8.** Calibration Plot for the CART tree model

# 1.0

Calibration

...intercept: −0.11 (−0.69 to 0.46)

...slope: 1.67 (0.86 to 2.48)

Discrimination

...c−statistic: 0.84 (0.75 to 0.93)

Ideal

Logistic calibration Flexible calibration (Loess)

1

0

0.8

0.6

Observed proportion

0.4

0.2

0.0

0.0 0.2 0.4 0.6 0.8 1.0

Predicted probability

**Supplemental Figure 9:** CrAg titer distribution among participants

**
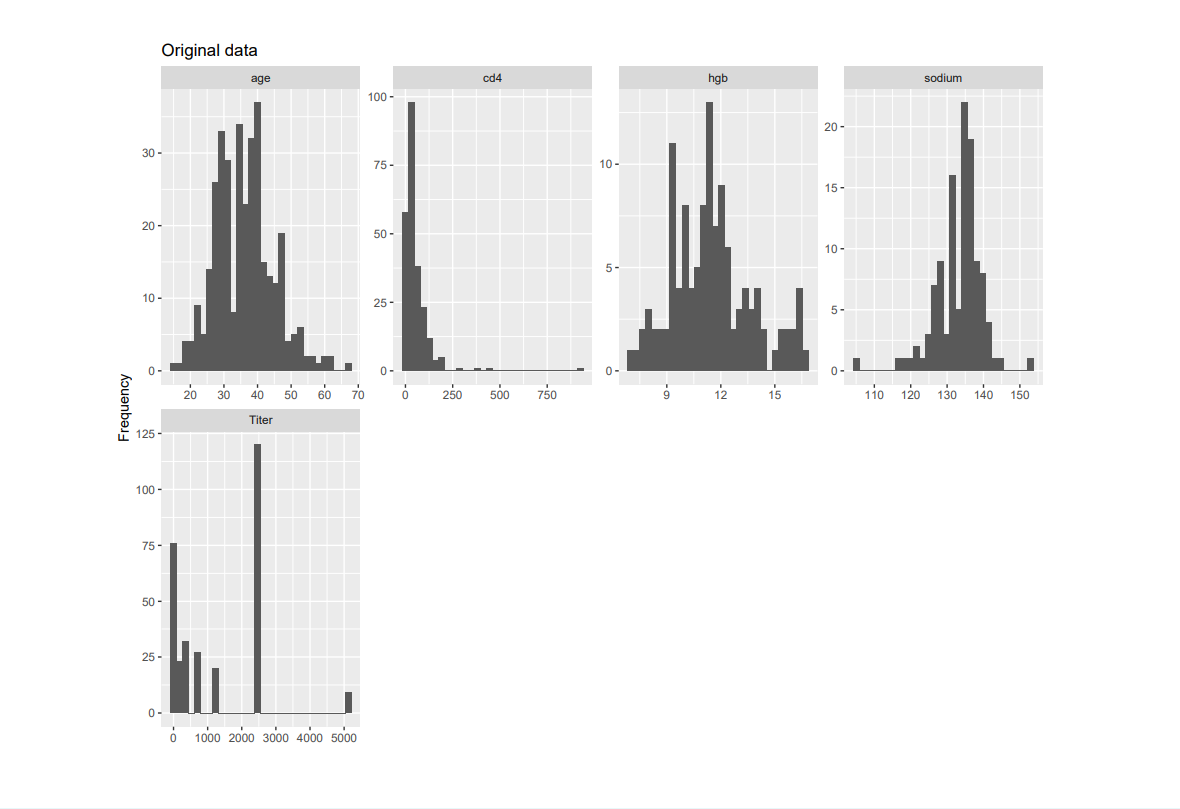
**
